# Anxiety Sensitivity Moderates the Association Between Father-Child Relationship Security and Fear Transmission

**DOI:** 10.3389/fpsyg.2020.579514

**Published:** 2020-10-14

**Authors:** Alexe Bilodeau-Houle, Valérie Bouchard, Simon Morand-Beaulieu, Ryan J. Herringa, Mohammed R. Milad, Marie-France Marin

**Affiliations:** ^1^Department of Psychology, Université de Montréal, Montreal, QC, Canada; ^2^Department of Psychology, Université du Québec à Montréal, Montreal, QC, Canada; ^3^Research Center of the Institut Universitaire en Santé Mentale de Montréal, Montreal, QC, Canada; ^4^Department of Neurosciences, Université de Montréal, Montreal, QC, Canada; ^5^Department of Psychiatry, University of Wisconsin School of Medicine and Public Health, Madison, WI, United States; ^6^Department of Psychiatry, New York University Grossman School of Medicine, New York, NY, United States

**Keywords:** observational fear conditioning, parent-child relationship quality, attachment, anxiety sensitivity, skin conductance response, parent-child dyads

## Abstract

Observational fear learning can contribute to the development of fear-related psychopathologies, such as anxiety disorders and post-traumatic stress disorder. Observational fear learning is especially relevant during childhood. Parent-child attachment and anxiety sensitivity modulate fear reactions and fear learning but their impact on observational fear learning has not been investigated. This study investigated how these factors contribute to observational fear learning in children. We examined this question among 55 healthy parent-child dyads. Children (8–12 years old) watched a video of their parent undergoing a direct fear conditioning protocol, where one stimulus (CS+Parent) was paired with a shock and one was not (CS−), and a video of a stranger for whom a different stimulus was reinforced (CS+Stranger). Subsequently, all stimuli were presented to children (without shocks) while skin conductance responses were recorded to evaluate fear levels. Our results showed that children more sensitive to anxiety and who had lower father-child relationship security levels exhibited higher skin conductance responses to the CS+Parent. Our data suggest that the father-child relationship security influences vicarious fear transmission in children who are more sensitive to anxiety. This highlights the importance of the father-child relationship security as a potential modulator of children’s vulnerability to fear-related psychopathologies.

## Introduction

Anxiety and fear-related disorders, such as post-traumatic stress disorder (PTSD), aggregate within families. Social transmission is recognized as an important mechanism of intergenerational transmission of anxiety (Eley et al., 2015). For example, observational fear learning has been shown to be a pathway leading to the development of phobias, particularly during childhood (Askew and Field, 2008). Moreover, fear responses learned within the familial environment might contribute to the intergenerational transmission of PTSD vulnerability (Bowers and Yehuda, 2015; Sherman et al., 2016). Hence, observational fear learning is relevant to better understand the processes contributing to the development of anxiety and fear-related psychopathologies.

Observational learning within the familial environment is important as children are sensitive to their parents’ emotions and rely on their reactions to respond to their environment (Murray et al., 2008). Studies have shown that mothers’ negative reactions to a stimulus or toward a stranger were associated with higher fear reactions in their children when they were confronted with the same stimulus or individual (Gerull and Rapee, 2002; de Rosnay et al., 2006; Egliston and Rapee, 2007; Murray et al., 2007; Dubi et al., 2008). In an observational conditioning protocol, pictures of unknown animals were presented alone or paired with static fearful or happy faces. Results showed that children’s subjective fear levels significantly increased for animals that were associated with the fearful faces (Askew and Field, 2007). A subsequent study found that children’s subjective fear levels were equivalent for animals associated with their mother’s fearful face and those associated with a stranger’s fearful face (Dunne and Askew, 2013). Also, in an observational fear learning protocol with parent-child dyads, children exhibited higher physiological fear levels to stimuli for which their parent or a stranger received a shock compared to a stimulus that was not reinforced. Mother-child dyads and father-child dyads showed similar physiological fear levels, suggesting that children can learn fear from both parents to the same extent (Marin et al., 2020a). Together, these results demonstrate that observational fear learning can be induced in laboratory settings and that children seem to learn as much from their parent than a stranger.

Since observational fear learning is important during childhood and may contribute to the development of fear-related psychopathologies, it is important to investigate which factors influence children’s sensitivity to observational fear learning within the familial environment. Parent-child attachment and anxiety sensitivity have both been shown to modulate one’s vulnerability for developing anxiety disorders and PTSD (McLaughlin et al., 2007; Kılıç et al., 2008; Colonnesi et al., 2011; Ogle et al., 2015). It is unclear, however, if these factors have any impact on children’s ability to observationally learn fear. Investigating these factors in healthy children could help to understand their contribution in fear-related disorders.

Parent-child attachment has been shown to modulate fear reactions in children. Studies have shown that when children are exposed to fearful, pleasant or neutral stimuli (e.g., film clips and images), an insecure attachment with the mother or both parents (using a combined measure) leads to higher physiological reactivity to the fearful stimuli (Gilissen et al., 2007, 2008; Stupica et al., 2017). While these studies found an impact of attachment on fear reactivity, they have not assessed whether attachment is involved in observational fear learning. Also, no studies on fear reactivity have specifically examined the contribution of the father-child relationship security.

The role of anxiety sensitivity on fear reactivity has been less studied. Studies have mostly examined the influence of anxiety sensitivity on physiological reactivity to a physiological stressor in adults and results have been mixed (Sturges et al., 1998; Zvolensky and Eifert, 2001; Spira et al., 2004; Conrod, 2006; Gregor and Zvolensky, 2008; Kelly and Forsyth, 2009). However, Gilissen and colleagues have found that the mother-child attachment influenced physiological fear reactivity only in children having a fearful temperament, a characteristic associated with anxiety (Schwartz et al., 1999; Biederman et al., 2001; Gilissen et al., 2007, 2008; Murray et al., 2009; Degnan et al., 2010; Rapee and Coplan, 2010). These results suggest an interaction between children’s anxiety and their attachment to their parents. Yet, the role of anxiety sensitivity and its interaction with mother-child and father-child attachment on fear learning remains to be determined.

We aimed to study the joint impact of parent-child relationship security and anxiety sensitivity on observational fear learning. As explained above, experimental protocols have shown that children learn fear equivalently when observing their parent or a stranger and that attachment influences children’s fear reactions to threat-related stimuli. Therefore, we hypothesized that an insecure relationship to the mother and the father would be associated with higher physiological fear levels, measured by skin conductance response (SCR), for threat-related stimuli, irrespective of whether the threat was signaled by the parent or a stranger. No effect of relationship security was expected for the safety-related stimulus. Also, given that Gilissen and colleagues found that the influence of attachment on fear reactivity was particularly important for more anxious children (fearful temperament), we expected a modulating role of the child’s anxiety sensitivity on the parent-child relationship security, where a negative association between parent-child relationship security and SCR would be present only for children more sensitive to anxiety.

## Materials and Methods

### Participants

Eighty-three French speaking biologically related parent-child dyads were recruited between August 2017 and April 2019, from a Canadian urban community through bulletin boards of some universities, local stores and community centers as well as social media and websites. Parents completed a telephone interview to ensure that they and their child were eligible. Inclusion age for children ranged from 8 to 12 years old. Exclusion criteria for parents were (i) history of bipolar or psychotic disorder, addiction or substance abuse; (ii) suffering from a severe or unstable medical condition; (iii) current use of psychiatric medication; and (iv) for mothers, pregnancy. Exclusion criteria for children were (i) history of mental health problems, developmental delays or brain damage; (ii) suffering from a severe or unstable medical condition; and (iii) history or current use of psychiatric medication. This study was approved by the local institutional review board and conducted in accordance with the Declaration of Helsinki. All parents provided written consent and all children signed an assent form before the beginning of the experiment. Parents received $70 for their participation while children received a $30 gift card.

### Procedures

#### Questionnaires

Parent-child relationship security was assessed with a validated French version of the Security Scale-Child Self-Report (Bacro, 2011). Children answered the questionnaire twice (once for the mother-child and once for the father-child relationship), even though they participated in the experiment with only one of their parents. Children had to choose between two statements the one most relevant to their relationship (e.g., “Some kids wish they were closer to their mom/dad BUT other kids are happy with how close they are to their mom/dad”) and then rated whether the selected statement was “sort of true” or “really true.” Items were scored on a 4-point Likert scale (1 = low security level, 4 = high security level). The security score was calculated by averaging the 15 items. The internal consistency for the French version is α = 0.76 for the mother and α = 0.82 for the father. The test-retest reliability for the French version is *r* = 0.73 for the mother and *r* = 0.88 for the father (Bacro, 2011). Children also completed a validated French version of the Childhood Anxiety Sensitivity Index (CASI) (Stassart and Etienne, 2014). This 18-item questionnaire measures sensitivity to the physiological symptoms of anxiety. Each item (e.g., “It scares me when I feel nervous”) is rated on a 3-point Likert scale. The total CASI score ranges from 18 to 54. A higher score indicates greater anxiety sensitivity. The French version has been validated in a non-clinical population and shows an internal consistency of α = 0.82 (Stassart and Etienne, 2014).

#### Fear Conditioning Protocol

The protocol used in this study has been developed and validated by Marin et al. (2020a). While the protocol takes place over 2 days, the current manuscript focused on fear conditioning, which happens on the first day of the protocol. Parents and children were tested in two different adjacent rooms.

##### Protocol for the parents

**FIGURE 1:**
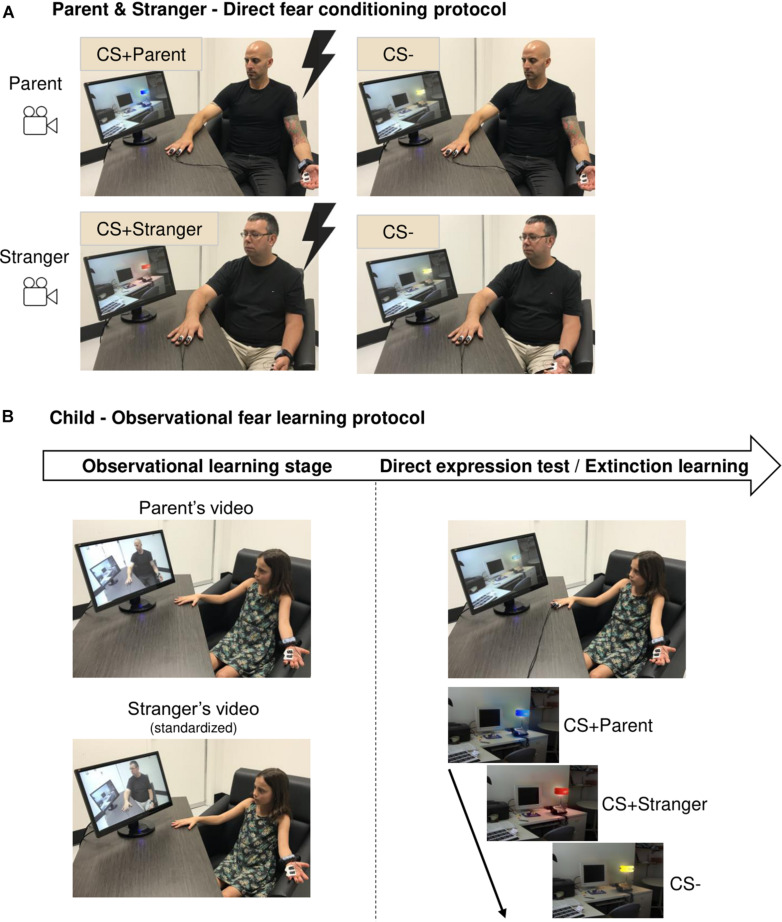
Summary of the protocol. **(A)** Direct fear conditioning protocol for the parent and the stranger. The parent was exposed to two colored lamps, one was paired with a mild electric shock (CS+Parent) and the other was not (CS–). A stranger adult was exposed to the same procedure, but a different colored lamp was paired with the shock (CS+Stranger). The CS– was the same than the parent. Both procedures were filmed. Lightning represents the administration of electrical stimulation. **(B)** Observational fear learning protocol for children. In the observational learning stage, children watched the videos of their parent and the stranger. Children were then exposed to the direct expression test and extinction learning, where the three stimuli (CS+Parent, CS+Stranger, CS–) were presented to them. They were instructed that they might receive a shock for some stimuli, but no shock was given to children in order to test observational learning.

Skin conductance recording Ag/AgCl electrodes were placed on the palm of the parents’ left hand and electrodes for electrical stimulation were placed on the right hand’s index and middle fingers. Parents selected a shock level that was highly annoying, but not painful (range: 0.8−6.0 mA). The protocol began with the habituation phase, where two colored lamps (e.g., blue and yellow lights) were presented twice and never ended with an electric shock. In the direct conditioning phase, one of the lamps (e.g., blue lamp) was presented eight times, with five of these presentations being paired with an electric shock (500 ms) delivered to the right hand’s second and third fingers [conditioned stimulus (CS+); CS+Parent]. The other lamp (e.g., yellow lamp) was presented four times without a shock [non-conditioned stimulus (CS−)]. Prior to the project, we recorded videos of two stranger adults (one man and one woman) who were exposed to the same procedure (habituation and direct conditioning) with the exception that the CS+ was different from the one used with the parent [e.g., a red lamp (CS+Stranger)]. The CS− was the same than the parent (e.g., a yellow lamp) (Figure 1A). Each trial was composed of a black screen (intertrial interval) lasting between 9 and 15 s (with an average of 12 s) followed by the image of an office with a lamp off that was presented for 3 s (baseline image). Then, the lamp went on (e.g., blue or yellow, CS) for 6 s. Reinforced trials ended with the administration of the shock. Parents were filmed during the direct conditioning phase (without audio). CS+ colors (red/blue) were counterbalanced across dyads.

##### Protocol for the children

Skin conductance Ag/AgCl electrodes were placed on the palm of children’s left hand. Children were then exposed to an observational fear learning protocol involving three phases: habituation, observational learning stage, and direct expression test/extinction learning. In the habituation phase, three colored lamps (blue, red, and yellow lights) were each presented twice to children. The observational learning stage consisted of the presentation, on a computer screen, of the parent’s and stranger’s videos. Viewing order of these videos was counterbalanced across children. The sex of the stranger was congruent with the accompanying parent’s sex. After watching both videos, the experimenter asked children which colored lamps were paired with a shock for the parent and the stranger and which colored lamp was never paired with the shock. This information was used to classify children as contingency aware or unaware. Following the observational learning stage, electrodes for electrical stimulation were placed on the index and middle fingers of children’s right hand. Children were told that they might receive a shock for some colored lamps, but no shock was given to children at any point. The direct expression test/extinction learning then occurred, where children were presented with the three stimuli (i.e., CS+Parent, CS+Stranger, CS−) on the computer screen, with eight presentations for each stimulus (intermixed presentation) (Figure 1B). As for their parent and the adult stranger, each trial was composed of a black screen (intertrial interval) lasting between 9 and 15 s (with an average of 12 s) followed by the image of an office with a lamp off (baseline image) that was presented for 3 s. Then, the lamp went on (e.g., blue, red, or yellow, CS) for 6 s. No trial was reinforced for children. Given that children never received a shock, we were expecting an extinction process to take place rapidly. Therefore, only the early part (the first two presentations of each CS) of the direct expression test/extinction learning phase was used to test fear acquisition assessed by children’s physiological fear levels.

##### Ethical considerations

Before each phase of the protocol, the experimenter reminded children that they had the right to withdraw from the experience at any point. The testing rooms were equipped with an intercom allowing children to communicate with the experimenter at any time. Also, to reassure children, the experimenter told them that if they wanted to stop the experiment, they could themselves remove the electrodes. The experimenter showed children how to remove these electrodes and had children tried doing it once before beginning the experimental procedure. Also, at the end of the experiment, we explained the purpose of the experiment to children and the reason why deception was used. At the end of the study, we did a short interview with the children to find out how they felt during the protocol.

#### Physiological Recordings and Data Processing

Electrodermal activity was recorded using BioNomadix wireless technology (BIOPAC, MP160) and AcqKnowledge software. To quantify children’s fear levels for each stimulus presentation, SCR was calculated by subtracting the mean skin conductance level during the 2 s preceding CS presentation (i.e., during the presentation of the baseline image) from the maximal skin conductance level during the CS presentation (e.g., blue lamp). Thus, SCR to the CS reflected changes in skin conductance beyond any change in skin conductance level produced by the baseline image. SCR data were square root transformed (Milad et al., 2007, 2008, 2009; Marin et al., 2016, 2017, 2020b). Data were screened for outlier values (+/− 3.29 standard deviations from the mean).

### Statistical Analyses

The impact of parent-child relationship security and children’s anxiety sensitivity on children’s physiological fear levels was analyzed with a multiple linear regression. This analysis was performed twice, once for the mother-child relationship security and once for the father-child relationship security. Our model included children’s SCR as the dependent variable, the relationship security with the other parent as a covariate (i.e., when testing for the mother-child relationship security, we included the father-child relationship security and vice versa) and the following predictors: stimulus (CS+Parent, CS+Stranger, CS−), parent-child relationship security, and anxiety sensitivity, as well as their interactions. Significant interactions were decomposed using the simple slope approach (Aiken et al., 1991).

## Results

### Sample Characteristics

Twenty-eight parent-child dyads of the initial 83 dyads were excluded from the analyses: technical difficulties resulting in a loss of SCR signal (*n* = 3), drop-out before the direct expression test/extinction learning phase (*n* = 1), no paternal figure and therefore no completion of the Security Scale-Child Self-Report questionnaire for the father (*n* = 4), incomplete data for the CASI (*n* = 1) and no awareness of the contingency (*n* = 19). Children who were not aware of the contingency were excluded based on results from previous studies showing that contingency awareness is necessary to observe differential SCR (higher SCR to the CS+ compared to the CS−) (Hamm and Vaitl, 1996; Lovibond and Shanks, 2002; Hamm and Weike, 2005; Tabbert et al., 2006, 2010, 2011). Analyses were thus conducted on 55 parent-child dyads: 16 mother-daughter dyads, 9 father-daughter dyads, 16 mother-son dyads, and 14 father-son dyads. The 55 parent-child dyads consisted of 51 parents (22 fathers and 29 mothers) and 55 children (30 boys and 25 girls) (four parents came to the lab with two children). Sample characteristics details are presented in Table 1. Mother-child relationship security and father-child relationship security did not differ [*t*(54) = 1.449, *p* = 0.153]. Parent’s selected shock level did not differ between mothers and fathers [*t*(49) = 1.125, *p* = 0.266].

**TABLE 1:** Demographic characteristics.

**Children**	
Sex	
Boys	30
Girls	25
Percentage Caucasians	76.4
Age	9.89 (1.46)
Anxiety sensitivity level	28.87 (5.92)
Father-child relationship security	3.14 (0.43)
Mother-child relationship security	3.23 (0.37)
**Parents**	
Sex	
Male	22
Female	29
Percentage Caucasians	89.8
Age	40.33 (4.66)
Education years	15.65 (2.55)
Shock level	
Male	2.46 (1.62)
Female	2.01 (1.26)

*Standard deviations are in parentheses.*

### Main Analyses

For the mother-child relationship security, the multiple linear regression revealed a significant effect of Stimulus [*F*(2,100) = 10.976, *p* < 0.001, ηp^2^ = 0.18], where children showed higher SCR to the CS+Parent (*M* = 0.674, *SE* = 0.062) and CS+Stranger (*M* = 0.720, *SE* = 0.056) relative to the CS− (*M* = 0.459, *SE* = 0.056) (both *p*_s_ ≤ 0.002). No other main effects or interactions were found (all *F*_s_ ≤ 1.636, all *p*_s_ ≥ 0.200) (Table 2).

**TABLE 2:** Main and interaction effects of mother-child relationship security and anxiety sensitivity in predicting physiological fear levels for each type of stimulus.

Predictor variables				

	*B*	*SE*	*p*	95% CI
**CS+Parent**				
(Constant)	0.673	0.062	<0.001	0.540, 0.792
Father-child security	–0.204	0.168	0.229	−0.540, 0.133
Mother-child security	0.215	0.206	0.303	−0.199, 0.635
Anxiety	–0.001	0.011	0.964	−0.021, 0.022
Mother-child security × Anxiety	–0.023	0.027	0.412	−0.077, 0.032
**CS+Stranger**				
(Constant)	0.720	0.056	<0.001	0.604, 0.832
Father-child security	0.101	0.152	0.510	−0.204, 0.406
Mother-child security	0.012	0.187	0.951	−0.362, 0.394
Anxiety	0.007	0.010	0.463	−0.011, 0.028
Mother-child security × Anxiety	–0.033	0.025	0.187	−0.082, 0.016
**CS−**				
(Constant)	0.459	0.056	<0.001	0.347, 0.577
Father-child security	0.103	0.152	0.502	−0.203, 0.409
Mother-child security	–0.127	0.187	0.500	−0.506, 0.253
Anxiety	0.007	0.010	0.473	−0.012, 0.027
Mother-child security × Anxiety	–0.006	0.025	0.817	−0.055, 0.044

**SE*, standard error; CI, confidence interval.*

**FIGURE 2:**
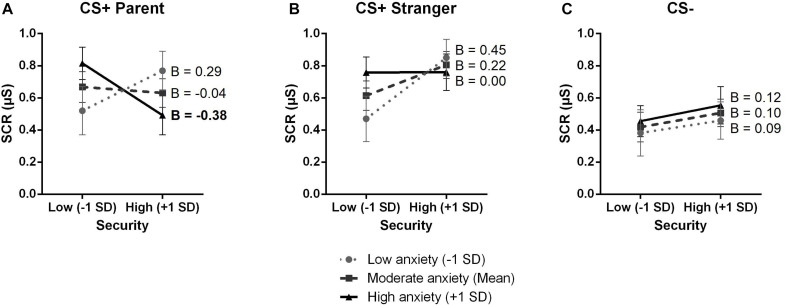
The effect of child’s anxiety sensitivity on the association between father-child relationship security and physiological fear levels as a function of stimulus. The *X* axis represents the score on the Security Scale-Child for the father and the *Y* axis represents children’s SCR, measured in microsiemens (μS). **(A)** Children’s anxiety sensitivity moderated the association between father-child relationship security and physiological fear levels for the CS+Parent. For children more sensitive to anxiety, lower security levels were associated with higher SCR. **(B)** Children’s anxiety sensitivity did not moderate the effect of father-child relationship security and physiological fear levels for the CS+Stranger. **(C)** Children’s anxiety sensitivity did not moderate the effect of father-child relationship security and physiological fear levels for the CS–. SCR, skin conductance response; *SD*, standard deviation. Error bars represent standard errors.

For the father-child relationship security, the multiple linear regression revealed a significant effect of Stimulus [*F*(2,100) = 10.319, *p* < 0.001, ηp^2^ = 0.17] and a significant Stimulus X Anxiety sensitivity X Father-child relationship security interaction [*F*(2,100) = 4.007, *p* = 0.021, ηp^2^ = 0.074]. The interaction between the father-child relationship security and anxiety sensitivity was significant for the CS+Parent (*F*(1,50) = 6.311, *p* = 0.015, ηp^2^ = 0.11) but not for the CS+Stranger and CS− (both *F*_s_ ≤ 3.104, both *p*_s_ ≥ 0.084) (Table 3 and Figure 2). Also, by correcting for the false discovery rate (Holm Hochberg), the effect remained significant for the CS+Parent (*p* = 0.0450, *p* = 0.131, *p* = 0.9170, for the CS+Parent, CS+Stranger and CS−, respectively). Simple slope tests showed that the effect of the father-child relationship security was significant when anxiety sensitivity levels were high (+1 *SD*, *B* = −0.3802, *p* = 0.035, 95% CI [−0.733, −0.027]), but not when they were moderate (Mean, *B* = −0.0438, *p* = 0.793, 95% CI [−0.377, 0.290]) or low (−1 *SD*, *B* = 0.2927, *p* = 0.239, 95% CI [−0.200, 0.786]) (Figure 2). More precisely, when anxiety sensitivity levels were high, a lower relationship security with the father was associated with higher SCR to the CS+Parent. No other main effects or interactions were found (all *F*_s_ ≤ 1.433, all *p*_s_ ≥ 0.244).

**TABLE 3:** Main and interaction effects of father-child relationship security and anxiety sensitivity in predicting physiological fear levels for each type of stimulus.

Predictor variables				

	*B*	*SE*	*p*	95% CI
**CS+Parent**				
(Constant)	0.646	0.060	<0.001	0.525, 0.765
Mother-child security	0.097	0.178	0.589	−0.260, 0.454
Father-child security	–0.044	0.166	0.793	−0.371, 0.300
Anxiety	0.001	0.010	0.939	−0.020, 0.021
Father-child security × Anxiety*	–0.057	0.023	0.015	−0.102, −0.011
**CS+Stranger**				
(Constant)	0.713	0.056	<0.001	0.599, 0.826
Mother-child security	–0.125	0.168	0.460	−0.462, 0.212
Father-child security	0.225	0.157	0.158	−0.087, 0.547
Anxiety	0.008	0.010	0.389	−0.011, 0.028
Father-child security × Anxiety	–0.038	0.021	0.084	−0.081 0.005
**CS−**				
(Constant)	0.467	0.057	<0.001	0.351, 0.581
Mother-child security	–0.144	0.170	0.401	−0.486, 0.198
Father-child security	0.104	0.159	0.518	−0.219, 0.425
Anxiety	0.007	0.010	0.470	−0.013, 0.027
Father-child security × Anxiety	0.002	0.022	0.921	−0.041, 0.046

*SE*, standard error; CI, confidence interval; *p ≤ 0.05.

## Discussion

We investigated whether the mother-child and the father-child relationship security as well as anxiety sensitivity had an impact on children’s physiological fear levels during an observational fear learning protocol. Our results revealed that the mother-child relationship security, the father-child relationship security and anxiety sensitivity did not separately influence children’s SCR for any of the three stimuli. However, anxiety sensitivity moderated the influence of father-child relationship security on children’s SCR when presented with the CS+Parent. A less secure father-child relationship was associated with higher SCR in children with high anxiety sensitivity levels, regardless of the sex of the accompanying parent.

We did not find an impact of the mother-child relationship security on children’s fear levels. This is inconsistent with previous studies showing that a secure relationship with the mother predicted lower SCR to fear-related stimuli (Gilissen et al., 2008, 2007). Yet, these studies did not have an associative learning component. Also, mother-child relationship security was found to impact fear levels only in children presenting a fearful temperament, a characteristic for anxiety proneness. Therefore, a secure relationship with the mother could be particularly beneficial for children with high anxiety levels. We did not find this moderating role of anxiety on the association between mother-child relationship security and physiological fear levels. Even if anxiety sensitivity and fearful temperament are characteristics for anxiety proneness, fearful temperament refers to traits such as shyness, fear and discomfort. It is possible that the differences between our measures explain these discrepancies in our results. Stupica et al. (2017) also reported that the parent-child relationship security had an impact on children’s physiological fear levels to threatening pictures. More than half of their sample had an insecure relationship with their parents. According to the clinical threshold for insecure attachment (Kerns et al., 1996; Bacro, 2011), only 22% of our sample had an insecure relationship with the mother and 26% with the father. This suggests that more children in our sample had a secure relationship with their parents compared to Stupica et al. (2017) study. In line with the findings by Gilissen et al. (2007, 2008), the parent-child relationship security might be especially beneficial for more vulnerable children (either by the nature of their temperament or their attachment relationship).

Consistent with this hypothesis, our results indicate that children more sensitive to anxiety were more vulnerable to the effect of an insecure parent-child relationship on physiological fear levels. However, while Gilissen et al., found this effect with the mother-child relationship security, our group found it only with the father-child relationship security. Yet, Gilissen and colleagues did not assess the father-child relationship security, so we cannot know to which extent the father-child attachment influenced their results. To the best of our knowledge, our study is the first to investigate the impact of the relationship with the father on children’s physiological fear levels. The fact that the father-child relationship security, and not the mother-child relationship security, was associated with fear levels for children more sensitive to anxiety might not be that surprising. Other studies have found an association between the relationship with the father and children’s anxiety. A study reported that the father-child attachment quality better predicted children’s anxious/withdrawal behavioral problems than the mother-child attachment quality (Verschueren and Marcoen, 1999). It was also found that at age 10, lower father-child attachment security and restricted maternal autonomy predicted higher anxiety levels at age 15 (Stuart Parrigon and Kerns, 2016). Studies have speculated that mothers and fathers have different roles on children’s socio-emotional development, given the different nature of their interactions with them. Mothers are thought to be involved in more nurturing, comforting, and caregiving activities, while fathers are thought to be more involved in playful activities (Bögels and Phares, 2008). Even if mothers can also be implicated in playful interactions with children, fathers are more turbulent, stimulating, and emotionally exciting for the child (Lamb, 2010). It has been suggested that this specific father-child interaction is particularly important for the child’s ability to compose with new situations and threats. Paquette (2004) proposed that through playful interactions with their children, fathers allow them to explore the environment (stimulation) while setting limits to ensure their safety (discipline). That way, children develop a sense of security and self-confidence, which promotes their socio-emotional development (Paquette, 2004). According to this theory, the father-child attachment could be better explained by the involvement of the father in playful interactions with the child (Kazura, 2000; Grossmann et al., 2002; Paquette, 2004; Newland et al., 2008). Dumont and Paquette (2013) assessed two dimensions of the father-child relationship: one using the Risky Situation, which is designed to assess stimulation and discipline, and one using the Strange Situation, which is designed to assess how children can be comforted. The authors found that the Risky Situation (which measures aspects of the parent-child relationship that are more characteristic of the father’s role) predicted children’s internalizing problems, while the Strange Situation (which measures aspects of the parent-child relationship that are more characteristic of the mother’s role) did not (Dumont and Paquette, 2013). Taken together, results of these studies demonstrated that the relationship with the father plays an important role in children’s anxiety. Our results suggest that the relationship with the father influences physiological fear levels in the context of observational fear learning. However, it is important not to interpret the present results as demonstrating that the father-child relationship security is more important than the mother-child relationship security in children’s anxiety and fear. Indeed, previous studies have found an effect of the mother-child relationship security on children’s anxiety and fear reactions (Buist et al., 2004; Roelofs et al., 2006; Gilissen et al., 2007, 2008; Bögels and Phares, 2008; van Eijck et al., 2012; Stupica et al., 2017). However, it surely highlights the importance of investigating children’s relationship with both parents separately, as they can have distinct influence on children’s anxiety and fear.

Another key finding from our study is that the mother-child and the father-child relationship security had no impact on the child’s SCR for the CS+Stranger and the CS−. Studies have reported no modulatory effect of the parent-child relationship security on fear levels to non-threatening stimuli (Gilissen et al., 2007, 2008; Stupica et al., 2017), which is consistent with our results for the CS−. However, we expected the parent-child relationship security to influence SCR to the CS+Stranger. In studies investigating the impact of the parent-child relationship on fear levels, a buffering effect of the mother-child attachment is typically found for non-specific threatening cues (such as movies or pictures). We therefore did not anticipate an effect that would be specific to the CS+Parent. Importantly, none of these studies have assessed the impact of attachment on a family-related cue. Our study is the first to investigate the influence of the mother-child and father-child relationships security on fear levels in an observational fear learning protocol where children observe their parent and a stranger directly exposed to an aversive situation. The mechanism explaining why the father-child relationship security only modulates fear responses to the CS+Parent warrants further investigation. Physiological synchrony is a potential mechanism that could explain the specificity of the effect. In fact, Pärnamets et al. (2019) found that higher synchrony of autonomic activity between a demonstrator and an observer facilitated fear learning for the observer. Physiological synchrony has been reported to be high between a parent and his/her child (Feldman, 2017). We have previously reported that the physiological synchrony between the parent’s SCR while undergoing the direct fear conditioning procedure and his/her child’s SCR while watching this procedure was predictive of the child’s SCR when directly exposed to the CS+Parent. Importantly, the synchrony between the stranger and the child did not predict the child’s response to the CS+Stranger, suggesting specificity (Marin et al., 2020a). Since only 23 children participated with their father in the current study, we did not assess how physiological synchrony could modulate the joint impact of father-child relationship security and anxiety sensitivity on fear levels.

The present study has limitations. First, the Security Scale-Child Self-Report does not allow to differentiate between an ambivalent or avoidant attachment to the parent. It has been suggested that an ambivalent attachment style is more associated with anxiety levels than an avoidant attachment style (Sroufe, 2003; Brumariu and Kerns, 2010; Colonnesi et al., 2011, but see Groh et al., 2017). Future studies should examine whether the different attachment styles would lead to different fear learning outcomes. In the same vein, our measure of attachment did not specifically assess the playful interactions between the child and his/her parent. Future studies should evaluate this dimension of the parent-child relationship and examine whether it has a key role in modulating fear levels. Second, while our main goal was to investigate the impact of the parent-child relationship security in general, it would have been interesting to examine whether results differ when children observe the parent with whom they had an insecure relationship. Such analyses were not possible since only 13 children came to the experience with a parent with whom they had an insecure relationship. Third, it is important to note that nearly a quarter of the sample did not understand the contingency. However, children who were not aware of the contingency tended to be younger, suggesting that the observational fear learning protocol used in this study was less suited for younger children. Removing the CS+Stranger could make the protocol simpler for younger children.

In sum, we showed that a more secure relationship with the father was associated with lower physiological fear levels in children more sensitive to anxiety when learning a fear association from their parent. This study highlights the importance of investigating the roles of both mother- and father-child relationships as well as children’s anxiety sensitivity when studying observational fear learning. Our results suggest that a secure relationship with the father may be a protective factor against the development of fear-related psychopathologies, especially for children having higher anxiety sensitivity. Our data could help to target children more sensitive to observational fear learning in the context of the familial environment and to guide preventive interventions for at-risk families. Interventions that specifically target the father-child relationship security could have a positive impact on the child’s fear levels, which could reduce the risk of developing fear-related psychopathologies.

## Data Availability Statement

The raw data supporting the conclusion of this article will be made available by the authors, without undue reservation.

## Ethics Statement

This study was reviewed and approved by the Comité d’Éthique de la Recherche du Centre Intégré Universitaire de Santé et de Services Sociaux (CIUSSS) de l’Est-de-l’Île-de-Montréal. Written informed consent to participate in this study was provided by the participants’ legal guardian/next of kin and obtained from the individual(s), and minor(s)’ legal guardian/next of kin, for the publication of any potentially identifiable images or data included in this article.

## Author Contributions

AB-H: conceptualization, formal analysis, investigation, writing – original draft, and project administration. VB: investigation and writing – original draft. SM-B: formal analysis and writing – review and editing. RH and MM: methodology and writing – review and editing. M-FM: conceptualization, funding acquisition, methodology, resources, supervision, and writing – review and editing. All authors contributed to the article and approved the submitted version.

## Conflict of Interest

The authors declare that the research was conducted in the absence of any commercial or financial relationships that could be construed as a potential conflict of interest.
